# Supplementary material for: Hybrid Models and Biological Model Reduction with PyDSTool
Source: PLoS Comput Biol. 2012 Aug 9;8(8):e1002628. doi: 10.1371/journal.pcbi.1002628 (PMC3415397; doi:10.1371/journal.pcbi.1002628)
Supplement: Text S4 — Complete source code for the PyDSTool package (version 0.88.120504). Includes API documentation and help files linking to web pages. This file is identical to the current public release on Sourceforge.net. (ZIP) [file pcbi.1002628.s004.zip › PyDSTool/html/PyDSTool.PyCont.TestFunc.Function-class.html]

xml version="1.0" encoding="ascii"?


PyDSTool.PyCont.TestFunc.Function


| Home | Trees | Indices | Help | PyDSTool |
| --- | --- | --- | --- | --- |

Package PyDSTool ::
Package PyCont ::
Module TestFunc ::
Class Function

[hide private]

[frames] | no frames]

# Class Function

source code

```
object --+
         |
        Function
```

Known Subclasses:
:   - AddTestFunction
    - , TestFunc
    - , DiscreteMap
    - , FixedPointMap

---

F: R^n --> R^m

Note: The function func needs to act on arrays, not numbers. Thus, in
the case where n=1, define the function as acting on a 1 dimensional
array, i.e. reference x[0] NOT x. This is so numjac works without having
to test cases (most generality).


|  |  |
| --- | --- |
| Instance Methods | [hide private] |

|  |  |
| --- | --- |
| \_\_init\_\_(self, (n, m), func=None, save=False, numpoints=None)  x.\_\_init\_\_(...) initializes x; see x.\_\_class\_\_.\_\_doc\_\_ for signature | source code |

|  |  |
| --- | --- |
| \_\_getitem\_\_(self, loc) | source code |

|  |  |
| --- | --- |
| \_\_setitem\_\_(self, loc, vals) | source code |

|  |  |
| --- | --- |
| \_\_call\_\_(self, \*cargs) | source code |

|  |  |
| --- | --- |
| findzero(self, X) | source code |

|  |  |
| --- | --- |
| diff(self, x0, ind=None) | source code |

|  |  |
| --- | --- |
| numhess(self, x0, ind1=None, ind2=None)  Computes second derivative using 2nd order centered finite difference scheme. | source code |

**Inherited from `object`**:
`__delattr__`,
`__getattribute__`,
`__hash__`,
`__new__`,
`__reduce__`,
`__reduce_ex__`,
`__repr__`,
`__setattr__`,
`__str__`


|  |  |
| --- | --- |
| Properties | [hide private] |

**Inherited from `object`**:
`__class__`


|  |  |
| --- | --- |
| Method Details | [hide private] |

|  |  |
| --- | --- |
| \_\_init\_\_(self, (n, m), func=None, save=False, numpoints=None)  *(Constructor)* | source code |

x.\_\_init\_\_(...) initializes x; see x.\_\_class\_\_.\_\_doc\_\_ for
signature

Overrides: object.\_\_init\_\_: *(inherited documentation)*

|  |  |
| --- | --- |
| numhess(self, x0, ind1=None, ind2=None) | source code |

Computes second derivative using 2nd order centered finite difference
scheme. MAKE MORE EFFICIENT IN FUTURE (i.e. when an index is in both ind1
and ind2)

Thus, for F: R^n ---> R^m, H is an (m,n1,n2) matrix, where n1, n2
are subsets of [1,...,n]

  


| Home | Trees | Indices | Help | PyDSTool |
| --- | --- | --- | --- | --- |

|  |  |
| --- | --- |
| Generated by Epydoc 3.0.1 on Fri May 4 15:24:07 2012 | http://epydoc.sourceforge.net |
